# Bioimpedance Vector Patterns according to Age and Handgrip Strength in Adolescent Male and Female Athletes

**DOI:** 10.3390/ijerph18116069

**Published:** 2021-06-04

**Authors:** Marcus Vinicius de Oliveira Cattem, Bruna Taranto Sinforoso, Francesco Campa, Josely Correa Koury

**Affiliations:** 1 Department of Basic and Experimental Nutrition, Nutrition Institute, State University of Rio de Janeiro, Rio de Janeiro 20550-900, Brazil; mv_cattem@hotmail.com (M.V.d.O.C.); bruna.taranto@nutricao.ufrj.br (B.T.S.); 2 Department for Life Quality Studies, University of Bologna, 47921 Rimini, Italy; francesco.campa3@unibo.it

**Keywords:** adolescent athletes, body composition, BIVA, confidence ellipses, fat-free mass, R-Xc graph, tolerance ellipses

## Abstract

Bioelectric Impedance Vector Analysis (BIVA) can be used to qualitatively compare individuals’ hydration and cell mass independently of predictive equations. This study aimed to analyze the efficiency of BIVA considering chronological age and handgrip strength in adolescent athletes. A total of 273 adolescents (male; 59%) engaged in different sports were evaluated. Bioelectrical impedance (Z), resistance (R), reactance (Xc), and phase angle (PhA) were obtained using a single-frequency bioelectrical impedance analyzer. Fat-free mass (FFM) and total body water were estimated using bioimpedance-based equations specific for adolescents. Female showed higher values of R (5.5%, *p* = 0.001), R/height (3.8%, *p* = 0.041), Z (5.3%, *p* = 0.001), and fat mass (53.9%, *p* = 0.001) than male adolescents. Male adolescents showed higher values of FFM (5.3%, *p* = 0.021) and PhA (3.1%, *p* = 0.033) than female adolescents. In both stratifications, adolescents (older > 13 years or stronger > median value) shifted to the left on the R-Xc graph, showing patterns of higher hydration and cell mass. The discrimination of subjects older than 13 years and having higher median of handgrip strength values was possibly due to maturity differences. This study showed that BIVA identified age and strength influence in vector displacement, assessing qualitative information and offering patterns of vector distribution in adolescent athletes.

## 1. Introduction

Strenuous training could be a matter for the competitive adolescent athletes, since high intensity and high training volume impose nutritional and functional risks to body development [1]. Exercise practice has been associated with the development of bone [2] and muscle tissues [3]. Fat-free mass (FFM) is considered a predictor of muscle strength and physical capacities [4,5,6,7]. Assessments of body composition contribute to verify the effects of physical activity and sports practice over time.

Muscle strength is another valuable measurement in physically active individuals as it impacts sports performance, daily activities, life quality and is related to low incidence and prevalence of diseases [8]. In order to assess handgrip strength, handgrip dynamometers are easy to use, simple, and not expensive [9]. Muscle strength is also related to gender, chronological and biological age, and body composition, since FFM is important to produce it and fat mass (FM) may limit it in contact sports, for example [10,11]. Handgrip strength has been used in youth soccer and female basketball players for talent identification [12,13].

Bioelectrical Impedance Analysis (BIA) can be used as a non-invasive method to estimate FFM, FM, and total body water (TBW) from electrical body proprieties of resistance (R) and reactance (Xc) while considering individual characteristics, such as sex, age, height, and weight [14,15]. BIA presents good correlation and concordance with dual energy X-ray absorptiometry (DXA) also when analyzing adolescent athletes [16]. However, BIA equations are dependent on specific characteristics of the population [15]. For this reason, in recent years, Bioelectric Impedance Vector Analysis (BIVA) has gained relevance for sports [17,18], because its qualitative and semi-quantitative analysis of impedance vectors and impedance components are directly plotted on the R-Xc graph. BIVA graphics are interpreted by impedance vector lengths and their ellipses and phase angle (PhA) [19]. PhA is derived from R and Xc, and it has been interpreted as an index of membrane integrity and water distribution between intra and extracellular compartments [20]. In addition, PhA has been used as a predictor of body cell mass, and for this reason, it has been employed as an indicator of nutritional status [21]. The complementary use of the BIVA and PhA may be helpful in the evaluation of changes of nutrition and hydration status in athletes [22].

Moreover, BIVA provides qualitative information of soft tissue classification and ranking, comparing individual vectors and ellipses to reference populations [23]. In this context, it is important to develop BIVA references for adolescent athletes considering handgrip strength. To the best of our knowledge, there are no studies that relate BIVA and handgrip strength in female and male adolescent athletes.

Considering the importance of body composition and strength to sports practice and for adolescent health, and considering BIVA a useful tool to assess adolescent athletes, the aim of this study was to analyze the efficiency of BIVA, considering chronological age and handgrip strength in female and male adolescent athletes.

## 2. Materials and Methods

### 2.1. Study Design and Subjects

This was a cross-sectional observational study. Two hundred and seventy-three Brazilian healthy adolescents (*n* = 161, males [59%]), aged mean 12.9 ± 0.9 years participated. All the data were collected at a sports-oriented public school located in the central region of the city of Rio de Janeiro, Brazil (2012–2013). This is an elementary full-time school that, unlike other public schools, offers 120 min of daily sports training and seven sports modalities: swimming, judo, badminton, athletics, soccer, volleyball, and table tennis, in which the students practiced different sports for the same amount of time.

The adolescents were classified as athletes, because they participated in training, skill development, and were engaged in competition, according to the definition described in Sports Dietitians Australia Position Statement: Sports nutrition for the adolescent athletes [24].

The participants were classified according to sex, handgrip strength (high—above median value; low—under median value) and chronological age (≤13 or >13 years). In adolescents, body composition is highly interrelated to biological maturity, due to hormones and growth factors function [1]. In the absence of consistent maturation indicators, adolescents can be divided into ≤13 and >13 years [25]. Mathias-Genovez et al. (2016) [26] showed that in the Brazilian adolescent population, 13 years was the age at which changes in body composition start due to biological maturation.

An a priori power analysis was conducted to determine the sample size using statistical software (G*Power v. 3.1.9.2, Stuttgart, Germany). The sample size calculation was performed assuming the values of *r* = 40%, α = 5%, and β = 20%, so the number of students estimated by each sex was 126. However, at the end of the study, 161 male and 112 female adolescents participated.

To participate in this study, adolescents and parents agreed to participate after a full explanation of the research objectives. This study was approved by the Ethics Committee of the Pedro Ernesto Hospital (CEP/HUPE 1.020.909).

### 2.2. Anthropometric and Body Composition Measurements

Weight was measured with a portable scale to the nearest 0.1 kg (Filizola, Brazil), height was measured with a stadiometer to the nearest 0.5 cm (Sanny, Brazil), and Body Mass Index (BMI = weight[kg]/height^2^[m]) was calculated.

BIA measurements were always performed in the morning, using a tetrapolar analyzer RJL (Quantum 101; Systems, Clinton Township, MI USA), which applies an alternating current of 800 μA at a single frequency of 50 kHz. Participants were in the supine position with a leg opening distant 45° from the median line of the body and the upper limbs distant 30° from the trunk. Electrodes were applied on the right wrist and ankle after cleansing the skin with alcohol in a thermo-neutral environment of 25 °C. To avoid disturbances in fluid distribution, participants were instructed to abstain from foods and liquids for at least 4 h as well as refrain from caffeine intake and intense physical activity 24 h prior to the BIA analysis. Before each testing session, the analyzer was checked with a calibration circuit of known impedance (resistance = 500.0 Ω; reactance = 0.1 Ω; 0.9% error). Resistance (R) and reactance (Xc) were used to calculate phase angle (PhA) [20]. FFM and total body water were assessed using a predictive equation developed by Horlick et al. [27]. The BIA predictive equations used in this study are listed in Table 1. Fat mass (FM) was calculated subtracting FFM from weight, and fat mass percentage was calculated by (FM/weight) × 100.

### 2.3. Handgrip Strength

Handgrip strength was assessed with a hand JAMAR-dynamometer (Asimow Engineering Co., Los Angeles, CA, USA) in both hands alternately, three times, and the mean value was recorded to obtain a single value of HG.

### 2.4. Bioelectrical Impedance Vector Analysis

BIVA was developed based on the R and Xc vectors normalized by height (H) [19,28]. The experimental data are plotted in the R-Xc graph and compared with the 95th-percentile confidence ellipses from a reference population. The correlation between R and Xc determines the ellipsoidal form of the bivariate probability distributions [28].

**Figure 1:**
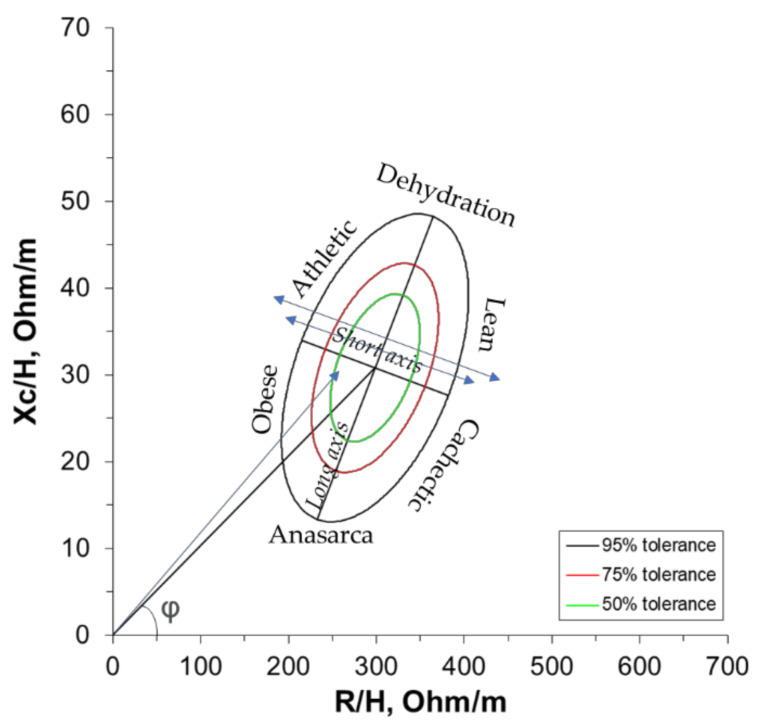
BIVA nomogram pattern, RXc-graph. Resistance (R) and reactance (Xc) were normalized by the height (H, meter) (adapted from Piccoli and Pastore, 2002).

BIVA tolerance consists of plotting the experimental data in a bivariate graph considering the 95th, 75th, and 50th vector percentiles of the Z-score of the reference population. Considering the plotting position of the experimental data, it is possible to suggest an interpretation: abnormal situation, when experimental data are positioned outside of the 95th percentile ellipsis; higher body cell mass, when experimental data are located above the long axis of the ellipsis; hypohydration, when experimental data are positioned to the right of the short axis of the ellipsis. Total body water is inversely related to the length of the impedance vector, and a combination of the vector length and its direction is defined as PhA [28,29] (Figure 1). The reference population for adolescents used in the BIVA analyses was obtained from the dataset of Koury et al. [16].

### 2.5. Statistical Analysis

All analyses were performed separately for each sex, and participants were classified according to chronological age (≤ 3 or >13 years) and handgrip strength median. Continuous variables were expressed as mean and standard deviation. An independent *t*-test followed by the Bonferroni post hoc test was used to compare variables between chronological ages. A linear regression model assessed the relation between handgrip strength (outcome) and chronological age, fat-free mass, and PhA (predictors). Univariate linear regression with backward stepwise elimination results were presented as unstandardized B coefficients, 95% confidence intervals (CI), and *p*-value. *p*-value < 0.05 was considered statistically significant. All statistical analyses were performed using STATISTICA 10 software (Stat Soft. Inc., Tulsa, OK, USA).

For BIVA, the two-sample Hotelling T^2^ test was used to compare differences in mean impedance vectors in BIVA confidence analyses, and the Mahalanobis test was used to calculate the distances between ellipses. Confidence and the 50%, 75%, and 95% tolerance ellipses were generated using BIVA software [29].

## 3. Results

Characteristics of the adolescent athletes according to sex and chronological age are shown in Table 2. Female adolescents showed higher values of R (5.5%, *p* < 0.01), R/H (3.8%, *p* = 0.041), Z (5.3%, *p* < 0.01), and fat mass (53.9%, *p* < 0.01) than male adolescents. Male adolescents showed higher values of FFM (5.3%, *p* = 0.021) and PhA (3.1%, *p* = 0.033) than female adolescents. According to chronological age, older female adolescents showed higher values of weight (19.9%, *p* < 0.01), height (3.2%, *p* < 0.01), BMI (13.5%, *p* < 0.01), PhA (5.1%, *p* = 0.002), FFM (14.9%, *p* < 0.01), FM (37.5%, *p* < 0.01), TBW (15%, *p* < 0.01), and handgrip strength (17.5%, *p* < 0.01). In addition to that, older female adolescents showed lower values of R (6.9%, *p* < 0.01), R/H (10.5%, *p* < 0.01), and Z (6.8%, *p* = 0.002) than younger participants. Older male adolescents showed higher values of weight (17.2%, *p* < 0.01), height (7.3%, *p* < 0.01), FFM (22.2%, *p* < 0.01), TBW (21.5%, *p* < 0.01), and handgrip strength (35.2%, *p* < 0.01); they showed lower values of R (7.5%, *p* < 0.01), R/H (15.3%, *p* < 0.01), Xc (8.9%, *p* < 0.01), Xc/H (16.4%, *p* < 0.01), and Z (7.7%, *p* < 0.01) than younger male adolescents. The different modalities practiced did not present any significant difference in the results of body composition and age.

**Figure 2:**
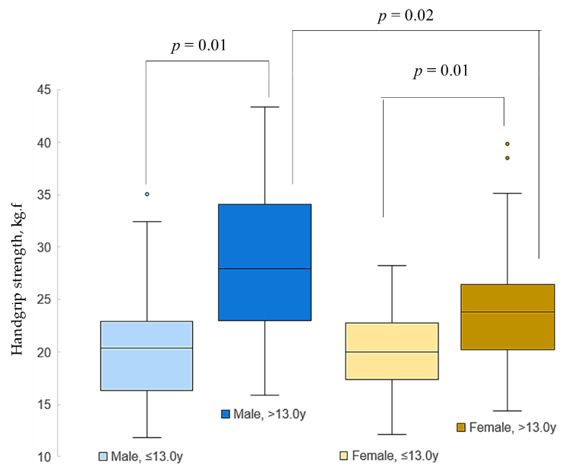
Handgrip strength in female and male according to different age classes (≤13 or >13 years).

Handgrip strength values are shown according to sex and chronological age (≤13.0 or >13.0 years) in Figure 2. The median value of handgrip strength was used to stratify female and male participants in groups of low and high handgrip strength. Individuals up to the median of handgrip strength of their sex were classified as low handgrip strength and individuals above the median were classified as high handgrip strength. The median of the female group was 20.6 kgf and that of the male group was 21.1 kgf. Differences were found between older and younger individuals of the same sex (*p* = 0.01) and between male and female participants at older age (*p* = 0.02), but not between younger subjects.

Table 3 shows that a linear regression model was applied to verify the influence of chronological age, FFM, PhA, and sex on handgrip strength (outcome). For all participants, chronological age (57.2%; *p* = 0.041) and FFM (62.2%, *p* = 0.0001) could explain the handgrip strength. In the female group, only FFM could explain the model in 56.1% (*p* = 0.0001), and in the male group, chronological age (79.2%, *p* = 0.032) and FFM (63.6%, *p* = 0.0001) could explain the handgrip strength.

**Figure 3:**
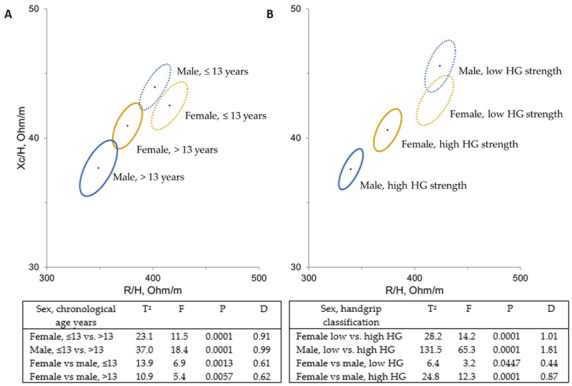
Mean impedance vectors with the 95% confidence ellipses for adolescent athletes sorted by chronological age (**A**) or handgrip strength classification (**B**). Mahalanobis distances (D), Hotelling T^2^-tests, F and *p*-values are included.

Figure 3 shows mean impedance vectors with 95% confidence ellipses for adolescent athletes according to sex and chronological age (Figure 3A) or sex and handgrip strength classification (Figure 3B). Participants showed differences when age and handgrip strength (*p* < 0.05) were compared. Older male and female athletes showed shorter impedance vectors. Similarly, a shorter impedance vector was observed in male and female participants with high handgrip strength. Additionally, when distances between age and handgrip strength ellipses were tested, a significant difference was found only between younger male participants and those with low handgrip strength (*p* = 0.033). In addition, there is a slight overlap in male and female low handgrip strength’ ellipses; however, the T^2^ test still found a significant difference. Considering age and handgrip strength, 35.6% and 33.7% of the younger female and male adolescents were classified as high handgrip (>median), and 44% and 23.3% of the older individuals were classified as low handgrip strength (<median), respectively.

**Figure 4:**
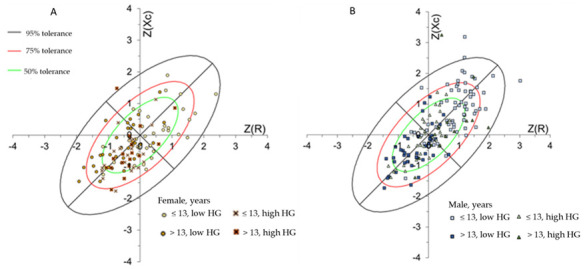
Mean impedance vectors with the 50, 75, and 95% tolerance ellipses for the female (**A**) and male (**B**) adolescent athletes, according to age and handgrip strength categories.

The data from female (Figure 4A) and male (Figure 4B) adolescent athletes, considering chronological age and handgrip strength classification, were plotted on the BIVA tolerance ellipses of Brazilian adolescent athlete reference population [16]. Both graphs presented a trend of a higher density of points in the 95% tolerance ellipsis. The frequency of points outside the 95% tolerance ellipsis, above the long axis, was 2% for male adolescents and 0.9% for female adolescent athletes. Only one female older and stronger subject was outside the 95% ellipse.

## 4. Discussion

There is a growing interest in BIVA in sports and physical exercise [17]. The present study shows, for the first time, BIVA patterns from female and male adolescent athletes and their associations with handgrip strength. Only FFM was a predictor of handgrip strength for female and male adolescent athletes. So, higher strength in male adolescents could be explained by the higher FFM throughout male development.

Studies in adolescent athletes are centered in male subjects [30,31,32]. There is only one study about BIVA in female athletes [23]. The present study is the first that shows BIVA responses associated with strength, brings new references for adolescent athletes, and adds knowledge to this field. Studies such as the present one, which assesses general health, are necessary in order to improve prescription of sports, since it is important to have information on adolescent athletes of both sexes.

Most studies only describe reference values for adult individuals, and thresholds and cutoffs points are needed for all ages and ethnic groups as reviewed by Dodds et al. [33] when analyzing variation in handgrip strength worldwide [33]. In the present study, handgrip strength did not show any statistical difference between female and male adolescents until the age of 13 years. However, it was greater in older male subjects than older female adolescents. In addition, female and male differences accentuated after 13 years of age, which may be attributed to puberty changes [34,35]. FFM/FM proportion may explain the greater strength in older male subjects. FFM is closely related to strength, since FFM is the primary body component that produces it [10]. However, when handgrip strength is standardized by fat-free mass, the difference disappears in this study dataset. Chronological age was important to discriminate male and female individuals by handgrip strength, but it was not a predictor in the linear model in female adolescents.

PhA is often associated with strength and physical fitness in adult athletes [18] and also in male adult and adolescent athletes [31]. PhA was also associated with handgrip strength in healthy adult men [36]. However, this study was conducted in an age range with little PhA variation according to a review of 250,000 subjects in different ages by Mattiello et al. [37]. For this reason, PhA could present a constant behavior in regression models and was not significant in all the analysis. Regarding the role of the somatic maturation on BIVA patterns, Campa et al. [2] identified specific transition periods in which the bioelectrical parameters showed an increase, a decrease, or a plateau. In particular, PhA begins to increase rapidly beginning at two years prior to the maturity offset and continues to do so for the four years following this growth phase [38]. In addition, the vector length shows a sharp decrease up to one year after the maturity offset, which is identifiable with the achievement of the peak height velocity, and then, it reaches a plateau. However, in athletes, the age at peak height velocity can be lower than that measured in the general population [30]. This may represent a common scenario in elite teams, as often there is a tendency to select taller athletes, which is typical in mature adolescents.

BIVA is an effective tool to assess body composition in male and female adult athletes [17,23], although there are no BIVA references to female adolescent athletes and no studies associating BIVA and handgrip strength in adolescent individuals.

In this study with adolescent athletes, BIVA confidence ellipses were sensitive both to age and handgrip strength. Confidence ellipses of older and stronger individuals shifted to the left, indicating increased cell mass and fluid content, which can be attributed to better cell functioning [17], which is consistent with growth development and physical training. It was also noticed that the ellipses of the female group had the same displacement in age and strength categorizations. Ellipses of the male group kept the same general pattern, but there was increased distance in strength categorization.

The hypothesis behind BIVA’s greater sensitivity to strength in male adolescents is related to maturity factors, in which the increasing strength is more relevant than chronological age. That means that strength reflects more the increase in body cell mass (especially FFM) and fluid content than age in male individuals. Although there is a slight overlap in both sexes’ ellipses in low strength groups, the Hotelling T^2^ test was able to identify a significant difference. Since confidence ellipses presented 95% probability, even a slight overlap could not affect the significance of Mahalanobis distance [17]. In this study, from the reference population, tolerance ellipses showed that most individuals were inside the 95% tolerance ellipses. The presence of female adolescents outside the ellipse may be explained by their better training status, which is reflected in higher cell mass; and male adolescents outside the ellipse may be explained by their hypohydration status expressed in long impedance vectors and reinforced by low total body water values (≤50% from weight).

A positive point of this study is a sample size (112 females and 161 males). Additionally, participants were measured in the same physical training conditions. These characteristics are particularly important to BIVA quality and applicability. Some limitations should be acknowledged. First, the present results refer to adolescent athletes and should not be generalized. Second, the bioelectrical parameters were measured using a foot-to-hand technology at 50 kHz frequency and should not be compared with the different technologies or data obtained at different sampling frequencies. Lastly, unfortunately, in the present study, it was not possible to assess the biological maturity status of the participants. However, our results are in agreement with other studies that used chronological age [26,34,39,40] and maturity status [32,38]. Deuremberg et al. [41] observed that a specific impedance was positively related with age until 13 years for both sexes, after which sex differences became apparent.

The assessment of BIVA patterns may assist in comparing adolescent athletes and identifying changes in body composition and the correlated hydration and cell mass qualitative information. BIVA identified the influence of age and strength in vector displacement. As the results show, handgrip strength may be an easier way to express biological maturity changes because of its correlation to FFM and how easy it is to be obtained. In fact, growth differences in female and male individuals are marked by the higher gain in FFM (and strength) in male than in female adolescents.

Handgrip strength is an acceptable indicator of overall muscle strength and health at any stage of life, from childhood to older age. BIVA is a promising alternative for assessing muscle strength, with potential application in other population groups.

## 5. Conclusions

The assessment of BIVA patterns may assist in comparing adolescent athletes and identifying changes in body composition and the correlated hydration and cell mass qualitative information. BIVA identified the influence of age and strength in vector displacement. As the results show, handgrip strength may be an easier way to express biological maturity changes, because of its correlation to FFM and how easy it is to be obtained. In fact, growth differences in female and male individuals are marked by the higher gain in FFM (and strength) in male than in female adolescents. Handgrip strength is an acceptable indicator of overall muscle strength and health at any stage of life, from childhood to older age. BIVA is a promising alternative for assessing muscle strength, with potential application in other population groups.

## Figures and Tables

**Table 1:** Predictive equations used in the present study.

Equations		Reference
Phase angle	=arc tangent (Xc/R) × (180°/π)	Baumgartner et al. [20]
Fat-free mass	=[3.474 + 0.459*H^2^/R + 0.064 × Wt]/[0.769 − 0.009*age − 0.016 × sex]	Horlick et al. [27]
Total body water	=0.725 + 0.475 × H^2^/R + 0.140 × Wt	Horlick et al. [27]

H = height (cm); Wt = weight (kg); R = resistance; Xc = reactance; sex = 0 for females and 1 for males.

**Table 2:** Descriptive and comparative general characteristics, according to sex and age categories (*n* = 273).

Characteristics	All			Age (Years)					
	Female	Male	*p*	Female		*p*	Male		*p*
				≤13.0	>13.0		≤13.0	>13.0	
*n*	112	161		59	53		101	60	
Age (years)	13.0 ± 0.9	12.8 ± 0.9	0.183	12.25 ± 0.46	13.82 ± 0.55	<0.01	12.28 ± 0.42	13.81 ± 0.50	<0.01
Weight (kg)	51.1 ± 10.1	48.9 ± 11.5	0.098	46.7 ± 9.9	56.0 ± 8.0	<0.01	45.9 ± 10.8	53.8 ± 10.9	<0.01
Height (cm)	157.7 ± 7.4	156.1 ± 9.9	0.153	155.3 ± 6.8	160.3 ± 7.2	<0.01	152.0 ± 7.9 **	163.1 ± 9.0	<0.01
BMI (kg/m^2^)	20.5 ± 3.4	19.8 ± 3.2	0.124	19.2 ± 3.1	21.8 ± 3.2	<0.01	19.7 ± 3.4	20.1 ± 2.8 **	0.446
R (Ω)	624.1 ± 70.2	591.7 ± 72.5	<0.01	643.8 ± 70.3	602.2 ± 63.8	<0.01	607.6 ± 72.6 **	565 ± 64.5 **	<0.01
R/H (Ω/m)	396.9 ± 50.4	382.2 ± 62.9	0.041	415.6 ± 51.8	376 ± 39.9	<0.01	402.0 ± 59.4	348.8 ± 54 **	<0.01
Xc (Ω)	65.7 ± 7.7	64.4 ± 9.2	0.230	65.8 ± 7.6	65.5 ± 7.8	0.836	66.4 ± 9.1	61.0 ± 8.5 **	<0.01
Xc/H (Ω/m)	41.8 ± 5.4	41.6 ± 7.6	0.851	42.5 ± 5.6	40.9 ± 5.0	0.125	43.9 ± 7.2	37.7 ± 6.6 **	<0.01
Z (Ω)	627.6 ± 70.2	595.8 ± 73.0	<0.01	647.2 ± 70.4	605.8 ± 63.9	0.002	612.2 ± 73.2 **	568.3 ± 64.5 **	<0.01
PhA (degree)	6.0 ± 0.7	6.2 ± 0.7	0.033	5.87 ± 0.6	6.24 ± 0.67	0.002	6.24 ± 0.67 ***	6.20 ± 0.83	0.746
FFM (kg)	38.9 ± 5.4	40.9 ± 8.2	0.021	36.3 ± 4.8	41.7 ± 4.5	<0.01	37.8 ± 6.6	46.2 ± 7.9 ***	<0.01
FM (kg)	12.2 ± 6.3	7.9 ± 6	<0.01	10.4 ± 5.9	14.3 ± 6.2	<0.01	8.1 ± 6.2 *	7.7 ± 5.8 ***	0.665
FM (%)	22.7 ± 8.3	15.2 ± 8.7	0.001	20.8 ± 8.1	24.8 ± 8.0	0.010	16.2 ± 9.0 ***	13.4 ± 7.9 ***	0.046
TBW (L)	27.1 ± 4.1	27.7 ± 5.9	0.371	25.3 ± 3.9	29.1 ± 3.3	<0.01	25.6 ± 5.0	31.1 ± 5.8 *	<0.01
HG (kgf)	21.0 ± 4.8	22.2 ± 6.5	0.110	19.4 ± 3.9	22.8 ± 5.1	<0.01	19.6 ± 4.9	26.5 ± 6.7 **	<0.01

BMI: body mass index; R/H: resistance/height ratio; Xc/H: reactance/height ratio; PhA: phase angle; FFM: fat-free mass; FM: fat mass; TBW: total body water; HG: handgrip strength. Intra- and intergroup differences were obtained using an independent *t*-test followed by the Bonferroni post-hoc test. Significant differences between sexes and the same age category were marked by * (*p* < 0.05), ** (*p* < 0.01), *** (*p* < 0.001).

**Table 3:** Handgrip strength independent predictive variables in adolescent athletes.

Variables	All *			Female			Male		
	β	95%CI	*p*-Value	β	95%CI	*p*-Value	β	95%CI	*p*-Value
Chronological age	0.572	0.024–1.119	0.041	0.109	–0.331–1.457	0.215	0.792	0.070–1.513	0.032
Fat-free mass	0.622	0.554–0.690	<0.01	0.561	0.429–0.694	0.001	0.636	0.559–0.714	<0.01
Phase angle	0.058	–0.117–1.087	0.114	0.093	–0.535–1.794	0.245	0.610	–0.093–1.313	0.089

Linear regression model. * adjusted by sex. R^2^ all = 0.651, R^2^ female = 0.386, R^2^ male = 0.753.
